# Designing Man’s New Best Friend: Enhancing Human-Robot Dog Interaction through Dog-Like Framing and Appearance

**DOI:** 10.3390/s22031287

**Published:** 2022-02-08

**Authors:** Ewart J. de Visser, Yigit Topoglu, Shawn Joshi, Frank Krueger, Elizabeth Phillips, Jonathan Gratch, Chad C. Tossell, Hasan Ayaz

**Affiliations:** 1 School of Biomedical Engineering, Science and Health Systems, Drexel University, Philadelphia, PA 19104, USA; yt422@drexel.edu (Y.T.); ha45@drexel.edu (H.A.); 2 Warfighter Effectiveness Research Center, United States Air Force Academy, Air Force Academy, Colorado Springs, CO 80840, USA; chad.tossell@academy.af.edu; 3 Department of Psychology, George Mason University, Fairfax, VA 22030, USA; fkrueger@gmu.edu (F.K.); ephill3@gmu.edu (E.P.); 4 Institute of Creative Technologies, University of Southern California, Los Angeles, CA 90007, USA; gratch@ict.usc.edu; 5 Department of Psychology, College of Arts and Sciences, Drexel University, Philadelphia, PA 19104, USA; 6 Drexel Solutions Institute, Drexel University, Philadelphia, PA 19104, USA; 7 Department of Family and Community Health, University of Pennsylvania, Philadelphia, PA 19104, USA; 8 Center for Injury Research and Prevention, Children’s Hospital of Philadelphia, Philadelphia, PA 19104, USA

**Keywords:** human-animal interaction, human-robot interaction, social robotics, Aibo, robotic features, behavioral analysis, social bonding, primacy effect, backstories

## Abstract

To understand how to improve interactions with dog-like robots, we evaluated the importance of “dog-like” framing and physical appearance on interaction, hypothesizing multiple interactive benefits of each. We assessed whether framing Aibo as a puppy (i.e., in need of development) versus simply a robot would result in more positive responses and interactions. We also predicted that adding fur to Aibo would make it appear more dog-like, likable, and interactive. Twenty-nine participants engaged with Aibo in a 2 × 2 (framing × appearance) design by issuing commands to the robot. Aibo and participant behaviors were monitored per second, and evaluated via an analysis of commands issued, an analysis of command blocks (i.e., chains of commands), and using a T-pattern analysis of participant behavior. Participants were more likely to issue the “Come Here” command than other types of commands. When framed as a puppy, participants used Aibo’s dog name more often, praised it more, and exhibited more unique, interactive, and complex behavior with Aibo. Participants exhibited the most smiling and laughing behaviors with Aibo framed as a puppy without fur. Across conditions, after interacting with Aibo, participants felt Aibo was more trustworthy, intelligent, warm, and connected than at their initial meeting. This study shows the benefits of introducing a socially robotic agent with a particular frame and importance on realism (i.e., introducing the robot dog as a puppy) for more interactive engagement.

## 1. Introduction

### 1.1. Robot Dogs Designed as Social Actors

Dog-like robots have become more commonplace both in people’s personal lives and in their workplaces. For example, the social robot Aibo can alleviate loneliness [1], serve as a companion [2,3], or be used as a tool for group therapy [4]. More recently, the Air Force adopted four-legged, dog-like robots with autonomous capabilities that can assist with patrolling and guarding remote parts of operational bases, thus freeing up personnel to focus on other tasks [5].

Dog-like robots are commonly designed and deployed as autonomous social actors because they are arguably one of the more successful social robot paradigms to date. The reason why this robot form is so effective may be because it is modeled after human-dog relationships, which exhibit effective communication, cooperation, attachment, and bonding [6]. Humans and real dogs have biologically co-evolved in mutually beneficial symbiotic relationships that have developed over centuries [7,8]. Thus, creating a social machine in dog form may foster more accurate mental models of trust in and bonding with these robotic companions [9,10] than other less familiar forms.

However, not all robotic dogs receive an equally warm welcome. Boston Dynamics Spot, a dog-like robot, received praise for its epic dancing moves online but when deployed in New York for street surveillance, caused concern among residents who were disturbed by these robots [11]. Depending on the context and use, the perception and trustworthiness of these autonomous “dogbots” can vary greatly and may need to be re-evaluated. Additionally, such systems could benefit from enhancing a robot dog’s “dog-likeness,” defined here as the degree to which an object is designed to mimic the form, characteristics, and behavior of a real dog.

The purpose of the current study was to investigate how enhancing the dog-likeness of a robot may improve human-robot interaction, mutual bonding, and trust. We used Sony’s newly released and improved version, the ERS 1000, of the Artificial Intelligence roBOt or Aibo (https://us.aibo.com/, accessed on 4 February 2022). Aibo is a social dog robot designed to be a partner, companion, and friend (Aibo means pal or partner in Japanese) and can learn and evolve with long-term use. In the mid-2000s, there was a wave of studies investigating earlier models like the ERS 110 and 210, but few studies to date have investigated the ERS 1000 [12,13]. This study expands on previous work by evaluating the new model and assessing the benefits of enhancing the dog-likeness of a robot through framing and appearance [14,15,16].

### 1.2. Improving the Dog-Likeness of the Aibo Robot Dog for Long-Term Bonding and Trust

Aibo was specifically designed to emulate a dog-like pet [17,18] and has been utilized as a platform for comparing human-animal interactions with human-robot interactions (HRI). Studies have shown that, compared to non-animal-like robots, animal-like robots such as Aibo lead to more interactive engagements due to their zoomorphic appearance and movements [12]. Previous research has further demonstrated that users express empathy towards Aibo and form trust relationships and long-term attachments with this robot [19,20,21,22,23,24,25,26]. For example, long-time owners of Aibo robots have shown extraordinary commitment and attachment to their robotic pets, demonstrated by persistent commitments to repair them and even holding funeral services for their pets once repairs could no longer be made [20].

Although Aibo is designed like a dog, research investigating the behaviors of people interacting with Aibo shows that a robotic dog is not the same as a real one. Even though Aibo is treated similarly to a real dog, people report enjoying interacting with a living dog more and doing so for longer [15,27,28,29,30,31]. Primary reasons why Aibo may not be loved as much as real dogs may be due to their lack of emotions, aliveness, and personality [32]. Thus, robot dog design and behavior, to date, has not sufficiently emulated a living dog.

**Figure 1:**
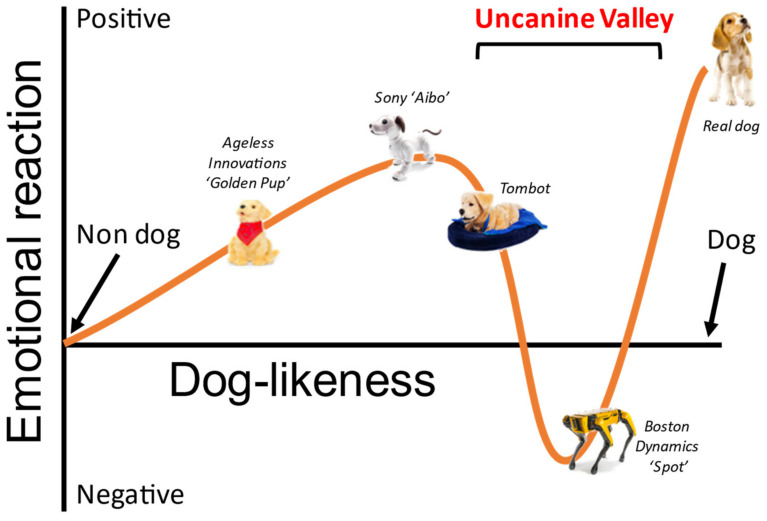
Representation of the “uncanine” valley, a hypothesized adaption of the uncanny valley. “Dog-likeness” is defined as the degree to which an object is designed to mimic the form, characteristics, and behavior of a real dog.

To address this design gap, Sony and researchers independently have tried to further improve Aibo’s dog-likeness by enhancing its appearance, behavior, and perception (see Figure 1). Improving on its original creation, Sony continuously re-designed Aibo’s form, and owners indicated that they preferred later generations of Aibo (ERS-7 and ERS-2xx) than the older generations [33]. Researchers have also found that enhancing an Aibo with puppy-scented fur stimulated adult dogs to approach the robot-like they approach a real puppy in some situations [34]. In terms of behavior, Aibo was enhanced with a complicated and unique behavior system that enables each Aibo robot to be unique in their internal “emotional states” [35]. Furthermore, extensive research has shown that framing the role, background, or origin of a robot can change how humans perceive and interact with it [36,37,38,39,40,41,42]. This may also be effective with Aibo as, for example, in Western society, dogs are already framed as “man’s best friend” [43]. Researchers have indeed shown that trust in Aibo can be increased by priming the initial expectations through framing Aibo’s role and agency when introducing the robot to participants [44]. People with higher initial expectations of a zoomorphic robot’s life-likeness may also be more likely to initiate companion-like relationships with the robot that are similar to human-human relationships [45].

Not all efforts to improve a robot’s dog-likeness may result in a positive outcome. A well-studied phenomenon in human-robot interaction is the uncanny valley hypothesis which predicts that as artificial agents become increasingly, but not perfectly human-like, peoples’ responses towards these agents become increasingly positive (e.g., likable, trustworthy) up to a point after which responses fall into a markedly negative “valley” (e.g., eerie, creepy) [46,47,48,49]. Recent studies have shown that there may also be an uncanny valley for robots that are animal-like (zoomorphic), such that robots high and low in animal-likeness (i.e., PARO, MiRO, etc.) were preferred over those mixing realistic and unrealistic features [50]. Recent news reports further indicate a potential public concern over creepy robot dogs [9]. The existence of an uncanny valley, or “uncanine valley” [13], for robotic dogs is, therefore, a possibility (see Figure 1), and attempts should be made to avoid it when enhancing the dog-likeness of a robot.

While most work defining human-likeness [51,52] or animal-likeness [50] has focused on the physical appearance and form of a robot, what comprises the overall assessment of a robot may include additional elements such as unique characteristics, personality traits, and behaviors. Recent work has begun to chart the relative contributions of these dimensions to outcome measures relevant to human-robot interaction [53,54]. Depending on the dog-like feature modeled on the x-axis (a combination of form, characteristics, behavior), the outcome measure (y-axis) may show a different pattern. In this paper, we focus on both the physical form of dog-likeness (Aibo’s shape and fur) and the behavioral component of dog-likeness by exploring how participants command Aibo and how it responds.

### 1.3. The Current Study

With Sony’s newly designed Aibo, the ERS 1000, we sought to re-evaluate its dog-like design and how this affects bonding, trust, and the potential for long-term interaction. Additionally, since previous studies have shown that enhancing the perception of Aibo can facilitate more engagement with the robot, we hypothesized the following:

**Hypothesis** **1** **(H1):** Participants will respond more positively to and interact more with an Aibo that is framed as a puppy (i.e., in need of development) rather than framed as simply a robot.

Because research has also shown that as a robot’s appearance approximates the intended agent it mimics (e.g., dog-like), it may result in more positive assessments of the robot, we also proposed the second hypothesis:

**Hypothesis** **2** **(H2):** Adding fur to an Aibo to increase its dog-like form will result in more positive assessments and interactions than an Aibo without fur.

We designed a 10-min study in which students from a military academy interacted briefly with an Aibo by issuing commands and engaging with the robot. To analyze human-robot interactions in detail, we used T-pattern analysis, a detection technique originally used by Magnusson to examine hidden real-time patterns in individual behavior [55]. This assessment method was used previously to compare individual behaviors of humans while interacting with an Aibo robot dog or with a real dog [15].

## 2. Materials and Methods

### 2.1. Participants

Twenty-nine participants (Age = 18–26 (20.53 +/−2.32 yrs); Females = 12) completed the study. All participants were recruited from the U.S. Air Force Academy and volunteered to participate in this study in exchange for course credits. For the analysis, one participants’ data were excluded due to poor quality in video recording.

### 2.2. Apparatus and Materials

**Figure 2:**
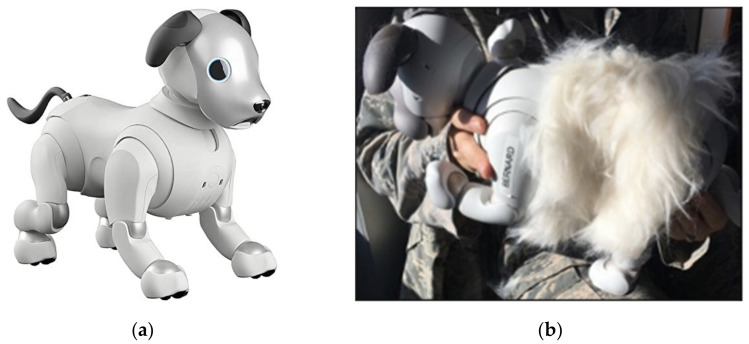
(**a**) Sony Aibo ERS-1000 without the fur; (**b**) Aibo outfitted with a fur suit.

Two ERS-1000 Sony Aibo dogs (Software version 2.5.1) were used for participant interaction in this study. The ERS-1000 has several cameras, sensors, and microphones that allow it to sense its environment (see Figure 2). Aibo also has two expressive OLED eyes that are used to convey expressions that the robot is tired, angry, or excited. Its ears, head, and tail can move to add further expressiveness and dog-like behavior. For the purposes of our experiment, we made one removable adaptation to Aibo by designing a fitted fur suit [13]. The suit was made from white textile faux fur that gave a realistic feel when touched and shed like a real dog. The fur was designed as a bodysuit on the dog and covered its back, shoulders, and hips. We attached the fur to the dog using Velcro. Along with the Aibos, we used a Lenovo ThinkPad laptop to video record the interactions that the participants had with the Aibos. Additionally, we used an open space, without any other people present, for the participants to interact with the dog. All questionnaires were administered to participants using Google Forms before and after the interaction (see Appendix B).

### 2.3. Task Paradigm

For easing the interaction between the participant and Aibo, the participants were given a list of commands that they could try with the Aibo. If the Aibo could understand the given command, Aibo would respond by engaging in behavior associated with the command. The commands and associated behaviors are shown in Table 1.

### 2.4. Video Coding and Self-Report Measures

For this study, each participant’s interaction with the Aibo was recorded with a video camera from a perspective that allowed for easy examination of both Aibo and participants’ actions. As shown in Table 2, the behaviors occurring throughout the interaction were categorized in terms of Aibo’s and individuals’ actions. After categorizing, two different experimenters manually coded footage second-by-second in each 10-min video to identify each unit of behavior and the start/end times for those behaviors for analyses. In the event of a coding mismatch, a third experimenter reviewed the footage again and made a final decision about the observed behavior in each case.

We used questionnaire assessments after a brief introduction of the robot dog and again after interacting with the Aibo to assess whether participants changed their opinions of the Aibo in a pre-post design. We asked participants to fill out a customized scale, developed in previous research [48], where participants rated their perceptions of Aibo across 12 characteristics. These measures are commonly used in studies of the uncanny valley effect, including creepiness, likability, scariness, trustworthiness, uncanniness, dog-likeness, consciousness, lifelikeness, intelligence, friendliness, connection, and warmth (see Appendix B for all 12 items). To establish clarity across participant ratings, we provided participants with definitions of each of the characteristics. Definitions were derived from definitions found in the Oxford English and Merriam Webster’s Dictionaries and Dictionary.com. Participants rated the Aibo both before and after interaction on each characteristic using Likert-type scales, which ranged from 0 (not at all) to 10 (extremely). Two additional items were included on this questionnaire as well; whether participants felt a connection with the dog and whether they thought the dog was warm and caring, rated from 1 (strongly disagree) to 5 (strongly agree).

### 2.5. Experimental Design and Procedure

For the experiment, we used a 2 × 2 between-subjects design with two independent factors: framing of the Aibo (puppy vs. robot), appearance (fur vs. no fur). Each of the 29 participants were randomly assigned to one of the four experimental conditions.

For framing, we framed Aibo either as a puppy or a robot when introducing Aibo to the participants. Two different scripts were developed for this purpose. In the puppy condition, Aibo was introduced by indicating that the dog was a puppy who was in training. We also used its name (Kipling or Bernard) and gendered pronouns like “he” and “him.” consistent with how previous research has introduced robots using backstories and framing [41,56,57,58]. In the robot condition, Aibo was described as a robot and was called, “Aibo” and referred to as “it” as a non-gendered identifier. The appearance condition consisted of Aibo either wearing a fur suit or not. In the fur condition, Aibo was outfitted with the fur suit (see Figure 2). In the no fur condition, the Aibo was presented to participants in its factory condition, without the fur suit. To differentiate these conditions, the name Kipling was used in the puppy no fur condition, while Bernard was used in the puppy fur condition.

For the study, participants began the experiment by completing an informed consent form. They were then randomly assigned into one of the four experimental conditions and guided to the experimental room, where participants were either introduced to an Aibo with a fur suit or without the fur suit. We then read the appropriate script either framing Aibo as a puppy with the name either Kipling or Bernard, or as a robot with the name Aibo. After a brief introduction to the dogbot, the participants then filled out the pre-interaction questionnaires about rating Aibo on the 12 characteristics mentioned. The participants were then instructed to interact with the Aibo robot for 10 min by attempting each of the eight commands provided in Table 1 with the robot. Throughout the experiment, a laptop was used to video record the participants interacting with Aibo. The experimenter left the room to allow participants to freely interact and attempt commands with Aibo. After 10 min, we asked the participants to fill out the post-interaction questionnaires, rating Aibo once again on the same characteristics. The entire experiment took approximately 20 min to complete.

### 2.6. Measures and Analysis of Commands Issued

Statistical analysis of the commands issued (including the total number of commands and command type) employed the use of linear mixed-effects with repeated measures across the entire sample, allowing for a population inference implemented in NCSS (NCSS, LLC. Kaysville, UT, USA; www.ncss.com, accessed on 4 February 2022), a comprehensive statistics software. The subject factor was treated as a random effect drawn from a larger population, while the fixed effects were conditions of framing and appearance. This analysis was employed to indicate any main or interaction effect of framing or appearance on command issuing behavior. The analyzed data is displayed in Table 3.

### 2.7. Measures and Analysis of Interaction Blocks

Interaction blocks within this experiment were defined as the initiation of a command (i.e., “Come Here”) ending with either the successful completion of the command by the Aibo, or the utterance of a different command (i.e., “Come Here” followed by “Sit Down” without completing “Come Here” first). The independent variables found within the Aibo and person were accounted for within interaction blocks, and linear mixed models with repeated measures were completed using the fixed factors of framing (puppy vs. robot) and appearance (fur vs. no fur) with subject as a random factor via the NCSS software. This modeling allowed for a higher resolution behavioral assessment compared to an overall interaction approach. Furthermore, this approach evaluated the duration of each person and Aibo. In addition to the Aibo, and person behaviors evaluated as dependent variables (see Table 2) using the linear mixed-effects approach.

### 2.8. T-Pattern Measures and Analysis

**Figure 3:**
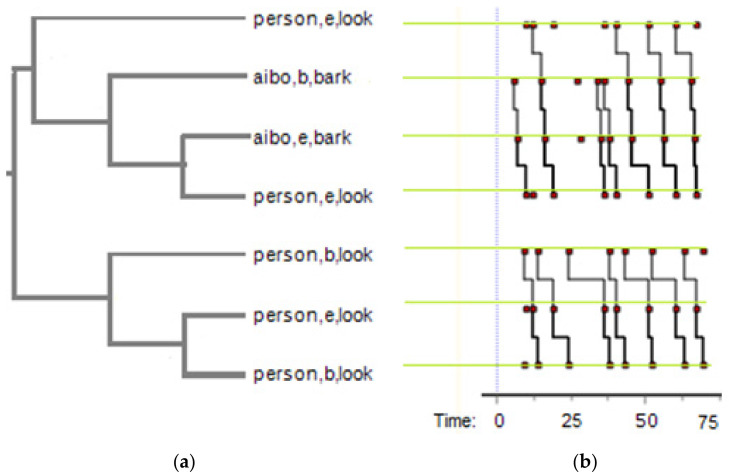
An example of an interactive T-pattern in ThemeEdu software; this is an example pattern extracted from one of the participants while interacting with Aibo. (**a**) The events occurring inside the specific pattern, listed in the order in which they occur within the pattern. The first event in the pattern appears at the top and the last at the bottom; (**b**) The frequencies of each behavior in the pattern, each red dot indicates a single behavior (e.g., the person begins looking, Aibo ends barking, etc.) at a certain time point. The light green lines on the red dots show the behavior which red dots represent. The black lines connecting the red dots represent the patterns between different behaviors. Smaller patterns inside the pattern can also occur when some of the events within the pattern occur without the whole pattern occurring.

T-pattern is a detection algorithm that is commonly utilized to discover the hidden or non-obvious time patterns in behavior [55,59,60,61]. The basic assumption of this methodological approach is that the temporal structure of a complex behavioral system is mostly unknown, however, this system may contain a set of particular types of repeated temporal patterns (T-patterns) composed of much simpler and more distinguishable event-types, which are coded in terms of their beginning and end points (e.g., “person, b, look” represents the behavior of “person begins looking at Aibo”, while “Aibo, e, bark” indicates “Aibo ends barking”, as can be seen in Figure 3a [15]. The set of time point series that results from such coding of behavior within a specific observation window acts as the input to the T-pattern definition and detection algorithms (e.g., the behaviors “person ends looking”, “Aibo starts barking”, “Aibo ends barking”, “person ends looking” in order creates a T-pattern, represented by the black lines between the red dots, as can be seen in case in Figure 3b).

For T-pattern analysis in our study, the interactions were transcribed and analyzed using ThemeEdu 6.0 software (www.patternvision.com, accessed on 4 February 2022) [62]. During the coding procedure, we recorded the start and end points of each action of both participant and Aibo. Taking our search for temporal patterns (T-patterns) into account, we used a minimum of three occurrences in the 10-min period for each pattern as a search criterion to filter out the non-repetitive patterns. The tests for the critical interval were set at *p* = 0.005. Using the software, we extracted the total amount of interactive behavior patterns, defined as the patterns that contain both the participant and Aibo “actors”. We extracted several measures, including the number of unique interactive patterns, the number of interactive pattern occurrences, the average complexity level, and length of the T-patterns in the 10-min period for each participant. For the number of unique interactive T-patterns, each different pattern inside the interaction was counted as one, while for the number of interactive pattern occurrences, we counted the total number of T-patterns inside the 10-min interaction. T-pattern complexity level refers to the number of behaviors inside a specific pattern subtracted by 1 (e.g., a T-pattern that contains four behavior units is a 3rd level T-pattern). Thus, more stacked levels of behavioral units result in a higher value for T-pattern complexity. We also extracted the total number of times that any of the behavior units for Aibo or the participant, listed in Table 2, occurred inside an interactive T-pattern. To analyze the extracted T-pattern features, we used linear mixed models with the framing (puppy vs. robot) and appearance (fur vs. no fur) as fixed factors and subjects as a random factor via NCSS software.

## 3. Results

### 3.1. Aibo Performance and Participant Command Behavior

When a command was issued, the Aibo had an acknowledgment accuracy of 81% (SE = 1.3%), responding with an acknowledgment bark in 5.94 s (SE = 0.27 s). The Aibo responded correctly 55% (SE = 1.7%) of the time within about 14.61 s (SE = 0.60 s). The Aibo completed the commands incorrectly (with a wrong response) 39% (SE = 1.6%) of the time, generally within 8.45 s (SE = 0.98 s). The Aibo did not respond to a command 9% (SE = 1%) of the time. 

We conducted the first analysis using a linear-mixed regression model involving commands issued (“Come Here” vs. “Very Lovely Aibo” vs. “Sit Down”, etc.) as a fixed factor with the participant as a random factor and Bonferroni corrections for post hoc analyses. Type of command issue was a significant factor (F_(7,216)_ = 20.522, *p* < 0.001***). Post-hoc analysis revealed that specifically, the “Come Here” task was issued at a significantly higher rate (between 5.75 and 7.39 additional issues) compared to any other task (*p* < 0.001*** for each post hoc comparison). No other command was issued at any further or lesser rate than the other.

Neither framing nor appearance led to any significant main or interaction effects regarding total numbers and types of commands issued (see Table 3). These nonsignificant findings indicate a similarity in the frequency and type of commands issued by participants across manipulations.

### 3.2. Interaction Block Results

**Figure 4:**
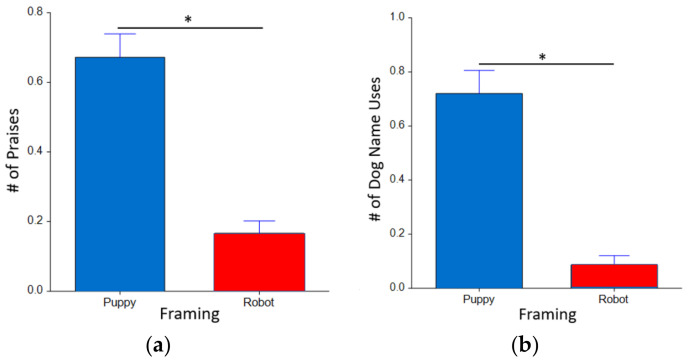
The average count of dog praises and dog name use across command blocks in each framing condition: (**a**) participants praised puppy Aibo more compared to robot Aibo; (**b**) participants used the dog name assigned significantly more while interacting with puppy Aibo, compared to robot Aibo.

For the statistical analysis of each behavior of the individual and Aibo in the interaction blocks, we used linear mixed models with framing (puppy vs. robot) and appearance (fur vs. no fur) as fixed factors with the subject as a random factor. As seen in Figure 4a,b, within the person behavior units, interaction blocks revealed a main effect for framing, revealing that when the Aibo was introduced as a puppy, participants called the Aibo by its dog name more often (F_(1,26)_ = 5.842, *p* = 0.023*, d = 0.670). Furthermore, in the puppy condition, Aibo’s were praised significantly more often (F_(1,25.8)_ = 6.807, *p* = 0.015*, d = 0.727). Aibo’s appearance as a factor was not significant neither in number of praises (F_(1,23.9)_ = 0.01*, *p* = 0.92) nor number of dog name uses (F_(1,24.0)_ = 0.53, *p* = 0.47).

### 3.3. T-Pattern Analysis

We used linear mixed models with framing (puppy vs. robot) and appearance (fur vs. no fur) as fixed factors to analyze T-pattern measures. Before the statistical analysis, we excluded the participants that had less than 100 interactive patterns and more than 10,000 interactions according to the T-pattern analysis to avoid outliers (4 participants). We compared the number of T-patterns and their complexity levels between each group of framing and appearance, in addition to how many times each behavior mentioned in Table 2 appeared in the interactive patterns.

**Figure 5:**
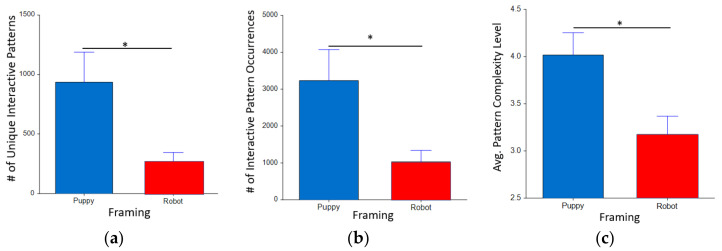
The number of interactive T-patterns and unique T-pattern interactions in each framing condition: (**a**) puppy Aibo led to more interactions compared to robot Aibo; (**b**) Interacting with puppy Aibo led to more unique interactive T-patterns compared to robot Aibo. (**c**) Interacting with puppy Aibo resulted in more complex interactive T-patterns compared to robot Aibo. * *p* < 0.05.

Participants interacted more with the Aibo when framed as a puppy both in terms of diversity of interaction (F_(1,20.0)_ = 4.88, *p* = 0.04*, puppy mean = 935.4 vs. robot mean = 262.8, d = 0.90) and quantity (F_(1,20.0)_ = 4.71, *p* = 0.04*, puppy mean = 3331.1 vs. robot mean = 1008.3, d = 0.87) of the interactive patterns, compared to Aibo introduced as a robot (see Figure 5). Furthermore, the interaction T-patterns with the Puppies were more complex on average (F_(1,20.0)_ = 7.25, *p* = 0.01*, d = 0.90) than robots. We did not find any significance for appearance in any of the T-pattern measures including total number of interactive T-patterns (F_(1,20.0)_ = 0.59, *p* = 0.49), number of unique T-patterns (F_(1,20.0)_ = 0.50, *p* = 0.49), and average T-pattern complexity (F_(1,20.0)_ = 0.13, *p* = 0.72).

**Figure 6:**
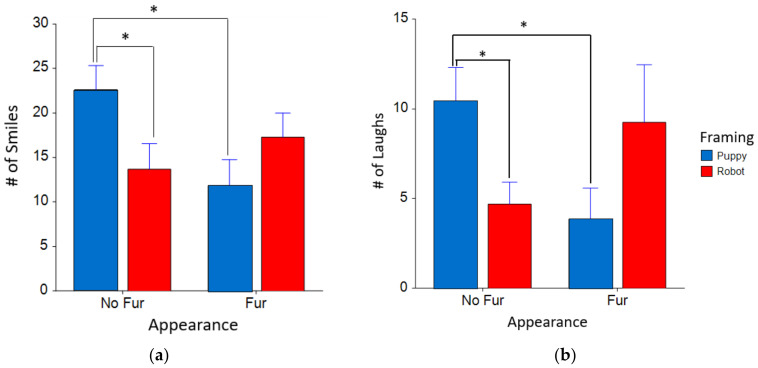
The number of times participants smiled and laughed based on Aibo behavior in interactive T-patterns across each framing and appearance condition. (**a**) Participants smiled at Aibo with puppy framing and no fur significantly more than puppy Aibo with fur and robot Aibo with no fur; (**b**) Participants laughed more while interacting with Aibo with puppy framing and no fur than puppy Aibo with fur; * *p* < 0.05.

The main interaction between framing and appearance was significant in terms of participants smiling (F_(1,20.0)_ = 5.75, *p* = 0.03*) and laughing (F_(1,20.0)_ = 7.45, *p* = 0.01*) in interactive T-patterns. Participants smiled significantly more in interactive T-patterns with the Aibo as a puppy with no fur, compared to Aibo as a puppy with Fur (F_(1,20.0)_ = 7.87, *p* = 0.01*, d = 1.48) and robot with no fur (F_(1,20.0)_ = 5.42, *p* = 0.03*, d = 0.96) (see Figure 6a). Participants laughed significantly more in the interactive T-patterns when the Aibo had no fur and was framed as a puppy, compared to puppy Aibo with fur (F_(1,20.0)_ = 6.26, *p* = 0.02*, d = 1.16) and robot Aibo with no fur (F_(1,20.0)_ = 4.77, *p* = 0.04*, d = 0.72) (see Figure 6b).

### 3.4. Questionnaire Results

To analyze the 12 questionnaire items, we used mixed linear mixed models with framing (puppy vs. robot) and appearance (fur vs. no fur) as the between-subjects factors and experience (pre vs. post-interaction) as a within-subject factor.

**Figure 7:**
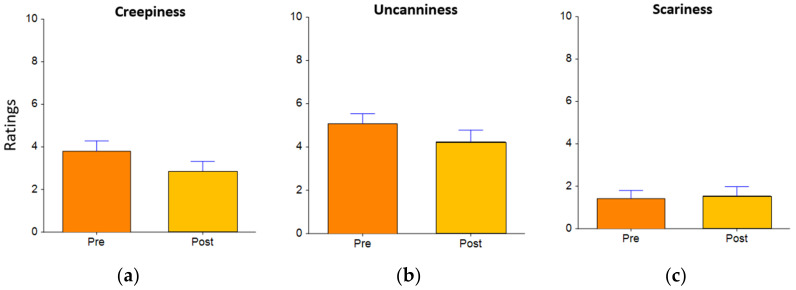
Self-reported ratings of questionnaires for “negative” characteristics (**a**) creepiness; (**b**) uncanniness, and (**c**) scariness) towards the robot pre-interaction (in orange) and post-interaction (in yellow).

**Figure 8:**
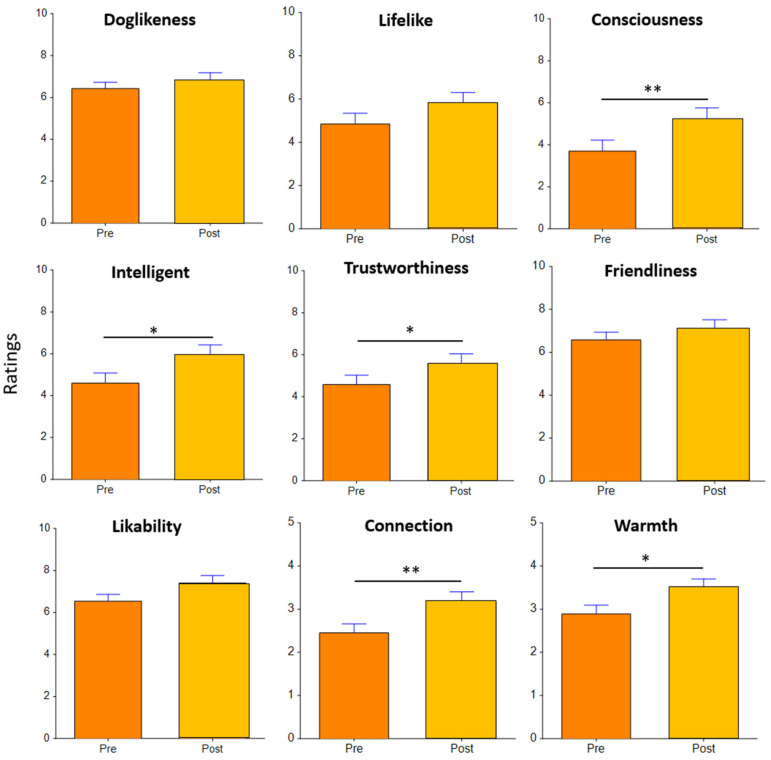
Self-reported ratings of “positive” perceived characteristics of the robot pre-interaction (in orange) and post interaction (in yellow). * *p* < 0.05, ** *p* < 0.01.

We did not find any significant effects of framing, appearance, or the interaction of both factors. Additionally, we did not find any significant interactions between pre-interaction and post-interaction ratings for the three “negative” characteristics, which are uncanniness, scariness, and creepiness (see Figure 7). However, we found that after interacting with Aibo, self-reported scores in terms of the robot’s “positive” perceived characteristics including trustworthiness (F_(1,24)_ = 5.02, *p* = 0.035*, d = 0.41; pre-mean = 4.57 vs. post-mean = 5.57), consciousness (F_(1,24)_ = 13.45, *p* = 0.001**, d = 0.53; Pre-mean = 3.70 vs. post-mean = 5.21), intelligence (F_(1,24)_ = 6.10, *p* = 0.02*, d = 0.52; pre-mean = 4.61 vs. post-mean = 5.93), connection (F_(1,24)_ = 9.08, *p* = 0.006**, d = 0.65; pre-mean = 2.45 vs. post-mean = 3.18) and warmth (F_(1,24)_ = 6.28, *p* = 0.019*, d = 0.59; pre-mean = 2.89 vs. post-mean = 3.50) significantly increased compared to the ratings before the interaction (see Figure 8).

## 4. Discussion

The purpose of the current study was to investigate how enhancing the dog-likeness of a dog-like robot may improve human-robot interaction, mutual bonding, and trust. After interacting with Aibo, participants felt Aibo was more trustworthy, intelligent, warm, and connected than at their initial meeting. While instructing Aibo, participants used the “Come Here” command most often. When framed as a puppy, participants used Aibo’s dog name more often, verbalized more praise, and exhibited more unique, interactive, and complex behavior with Aibo. Framing and appearance also interacted. Participants exhibited the most smiling and laughing behaviors with Aibo framed as a puppy with no fur compared to a puppy with fur or a robot with no fur. The strong framing is consistent with previous human-robot interaction studies, which have shown that personification of a robot improves people’s engagement and positive perceptions of that robot [36,37,38,39,40,41,56].

Interestingly, adding fur inhibited social reactions like smiling and laughing when Aibo was framed as a puppy, contrary to Hypothesis 2, which posed that adding more dog-like fur would enhance perceptions of dog-likeness and improve overall human-dog-robot interaction. One explanation may be that the fur suit did not cover the entire Aibo, but only part of its body. This may have resulted in a somewhat “uncanine” effect [50], although this was not reflected in subjective reports. Furthermore, the use of fur did not reduce social reactions when the Aibo was framed as a robot, indicating tolerance amongst users for realistic additions to robots. It is possible that with the framing of the Aibo as a robot, expectations for matching the exact fidelity of a dog were lower compared to the puppy frame. Because possible mechanisms of uncanniness are routed in perceptual mismatches [49,63], the mismatch between the robot frame and the fur may have been much lower or non-existent compared to the other conditions.

Given the design iterations of Aibo, it is possible that co-evolution between humans and robot dogs is already underway [9]. The new version of Aibo elicits a sufficiently interactive response from human partners due to its dog-like form and basic behaviors. Yet, the Aibo does not always respond accurately or speedily and may not adapt as fast as a real dog, providing a potential limitation for longer-term bonding and trust development beyond the initial interaction [64,65,66]. For example, previous work has indicated that robots with very basic abilities are eventually abandoned after a few months [67]. On the other hand, early task successes with robots can reinforce people to form positive attitudes towards their interactions [12]. Newer and updated versions of the Aibo may thus need to focus on improving these performance characteristics.

There were several limitations with this study regarding the robot dog, the participants, and measurements, which can be improved in future explorations. First, we only used the most recent version of Sony’s Aibo dog. Other appearance configurations could be examined by comparing the Aibo to previous versions, the Spot, toy dog robots, other companion dog robots, or real dogs. The fur could also be made more realistic, cover the entire robot body and take on additional colors and textures. The different name use of Kipling or Bernard in the fur conditions for puppy framing could have contributed to some differences across those conditions, although recent research suggests that name use has a negligible effect on outcome measures in human-robot interaction [59]. Additionally, all interactions with Aibo were brief—future research could explore long-term interactions with Aibo across several days or weeks. Other populations could be explored, including different age groups, robot dog owners, civilians, and military personnel. If robotic dogs are deployed more widely in a community, by the police, for example, additional design considerations may be needed to accommodate the composition of an entire community, including children, adults, and even pets like dogs [68], as each member may respond to the robot in a unique way. Furthermore, two-way interactions are critical for bonding between humans and dogs, such as when police or military personnel and canines work together to find explosives or when a blind handler works with their guide dog to navigate [8]. Studying more interactive, physical, and ecologically valid behaviors could be beneficial, including playing fetch, tug-of-war, games, or common work tasks. Measurements could have been improved by recording data directly from Aibo and by automatic classification of emotions using facial recognition software. Finally, while the study could have had a larger study population, our approach mitigated potential concerns about analyzing a small sample, including reporting moderate to large effect size for all effects, analyzing multiple repeated measures for each participant, and the use of linear mixed-effects modeling, which is an optimal analytical method to use when sample sizes are smaller.

These limitations notwithstanding, our study showed that the Aibo platform and its dog-like form continue to be a successful interaction paradigm for human-robot interaction as evidenced by the increased connection, warmth, and trustworthiness felt before and after only a brief interaction with the Aibo ERS-1000.

## 5. Conclusions

Our study demonstrated that framing a dog-like robot as a learning puppy can enrich behavioral interactive patterns and perceptions between humans and robots. Future research could focus on developing unique robot dog framing depending on the collaborative working environments and situation with humans while executing collaborative activities such as guarding, herding, navigating, detecting explosives, and conducting urban search and rescue operations. Creating frames based on the demands of the context may further foster the co-evolution between humans and robot dogs and sustain long-term human-robot engagement and bonding.

## Figures and Tables

**Table 1:** Commands and their corresponding behaviors for Aibo.

Command	Behavior
“Very Lovely Aibo”	Dances and barks as a song plays
“Sit Down”	Sits down in dog-like posture and pants
“Take a Picture”	Counts down and snaps a picture with a camera
“Sing a Song”	Strikes a sitting pose and sings a tune
“Come Here”	Turns and walks towards the speaker
“Happy Birthday”	Dances and barks as “Happy Birthday” song plays
“Let’s Pose”	Rolls over on belly and moves feet
“If You’re Happy and You Know it”	Dances and barks to the famous song

**Table 2:** Behavioral units recorded within the video per and between Aibo and participant.

Aibo’s Behavior Units	Person’s Behavior Units
Wags Tail	Laughs
Rotates Body	Smiles
Rotates Head	Pets Aibo
Kneels	Praises Aibo
Sits	Relocates Aibo
Barks	Repeated Commands
Looks at Person	Looks at Aibo
Lays Down	
Pivots Back and Forth	

**Table 3:** Commands issued totals per command types, and per framing and appearance factors.

Commands Issued	Overall (n = 29)	Robot (n = 11)	Puppy (n = 18)	No Fur (n = 15)	Fur (n = 14)
“Very Lovely Aibo”	2.14 (0.26)	2.19 (0.42)	2.08 (0.31)	2.52 (0.32)	1.75 (0.41)
“Sit Down”	3.46 (0.67)	2.79 (1.08)	4.13 (0.79)	2.79 (1.05)	4.21 (0.82)
“Take a Picture”	2.07 (0.33)	2.02 (0.53)	2.12 (0.39)	2.39 (0.41)	1.75 (0.52)
“Sing a Song”	3.28 (0.52)	3.21 (0.85)	3.35 (0.62)	3.06 (0.65)	3.5 (0.83)
“Come Here” ***	9.08 (1.36)	8.33 (2.20)	9.83 (1.61)	9.12 (1.68)	9.04 (2.15)
“Happy Birthday”	2.40 (0.52)	1.88 (0.83)	2.92 (0.61)	2.79 (0.64)	2.00 (0.81)
“Let’s Pose”	2.05 (0.36)	1.71 (0.45)	2.38 (0.33)	2.09 (0.34)	2.00 (0.44)
“If You’re Happy and You Know it”	2.02 (0.36)	1.75 (0.58)	2.29 (0.43)	2.25 (0.45)	1.79 (0.57)
Total	27.20 (2.82)	24.17 (4.55)	30.24 (3.34)	29.37 (3.48)	25.04 (2.82)

Values indicated are mean (standard error; SE) frequency repeats per experimental session, further split in either the framing of appearance conditions. *** indicates that “Come Here” was utilized at a significantly higher rate than any other command (*p* < 0.001 ***), while all the other commands were issued at similar rates compared to another.

## Appendix A. The Scripts for Puppy and Robot Conditions

Puppy Script: Today, you will be playing with (Bernard/Kipling). He is a robotic puppy who is still in training. He is only a puppy, so please be patient with him because he is still learning obedience. He might take a second to warm up because he is a little shy. The playdate today will be recorded and reviewed for future reference and data collection. Past researchers have found that playing with these dogs like (Bernard/Kipling) reduces stress. You will play with (Bernard/Kipling) for 10 min to try out different commands. Please try the following commands”:

“Very lovely Aibo”; “Sit down”; “Take a picture”; “Sing a song”; and “Come here”.

But after that, you can play with him however you please, in a respectful manner, of course. After you’re done playing with (Bernard/Kipling), you will be given a post-test. Please fill this test out thoroughly. Thank you and have fun! If you have any questions, please let us know.

Robot Script: Today, you will be interacting with Aibo. It is a robot whose actions and manners are similar to that of a dog. With the software programmed into Aibo, it might take some time to recognize your voice. The interaction today will be recorded and reviewed for future reference and data collection. Past researchers have found that playing with robots like Aibo can reduce stress. You will interact with Aibo for 10 min to try out different commands. Please try the following commands”:

“Very lovely Aibo”; “Sit down”; “Take a picture”; “Sing a song”; and “Come here”.;

But after that, you may interact with it however you please, in a respectful manner, of course. After you are done with Aibo, you will be given a post test. Please fill this test out thoroughly. Thank you, and if you have any questions, please let us know.

## Appendix B

1. Creepiness is “the characteristic of causing an annoyingly unpleasant sensation”. Please rate this robot on a scale of 0 (not at all creepy) to 10 (extremely creepy).
2. Likability is “having qualities that cause others to feel warm familiarity, comfort, and favorable regard”. Please rate this robot on a scale of 0 (not at all likable) to 10 (extremely likable).
3. Scary is “the characteristic of causing fright or alarm”. Please rate this robot on a scale of 0 (not at all scary) to 10 (extremely scary).
4. Trustworthy is “the characteristic of inducing a sense of being reliable, capable, ethical and sincere”. Please rate this robot on a scale of 0 (not at all trustworthy) to 10 (extremely trustworthy).
5. Uncanny is “the characteristic of seeming mysterious, weird, uncomfortably strange or unfamiliar”. Please rate this robot on a scale of 0 (not at all uncanny) to 10 (extremely uncanny).
6. Dog-likeness is “the characteristic of having the appearance and traits of a dog”. Please rate this robot on a scale of 0 (not at all dog-like) to 10 (extremely dog-like).
7. Conscious is “the characteristic of being aware of one’s own existence, sensations, thoughts, and surroundings”. Please rate this robot on a scale of 0 (not at all conscious) to 10 (extremely conscious).
8. Lifelike is “the characteristic of being able to adapt to one’s environment”. Please rate this robot on a scale of 0 (not at all lifelike) to 10 (extremely lifelike).
9. Intelligent is “the characteristic of having good understanding or a high mental capacity; quick to comprehend”. Please rate this robot on a scale of 0 (not at all intelligent) to 10 (extremely intelligent).
10. Friendly is “the characteristic of being like a friend; kind, helpful”. Please rate this robot on a scale of 0 (not at all friendly) to 10 (extremely friendly).
11. I felt I had a connection with the robot (Use scale from 1 (Strongly Disagree) to 5 (Strongly Agree)
12. The robot was warm and caring (Use a scale from 1 (Strongly Disagree) to 5 (Strongly Agree).
